# Genesis of a Fungal Non-Self Recognition Repertoire

**DOI:** 10.1371/journal.pone.0000283

**Published:** 2007-03-14

**Authors:** Mathieu Paoletti, Sven J. Saupe, Corinne Clavé

**Affiliations:** Laboratoire de Génétique Moléculaire des Champignons, UMR-5095 Centre National de la Recherche Scientifique (CNRS) et Université Bordeaux 2, Institut de Biochimie et Génétique Cellulaires (IBGC), Bordeaux, France; University of Queensland, Australia

## Abstract

Conspecific allorecognition, the ability for an organism to discriminate its own cells from those of another individual of the same species, has been developed by many organisms. Allorecognition specificities are determined by highly polymorphic genes. The processes by which this extreme polymorphism is generated remain largely unknown. Fungi are able to form heterokaryons by fusion of somatic cells, and somatic non self-recognition is controlled by heterokaryon incompatibility loci (*het* loci). Herein, we have analyzed the evolutionary features of the *het-d* and *het-e* fungal allorecognition genes. In these *het* genes, allorecognition specificity is determined by a polymorphic WD-repeat domain. We found that *het-d* and *het-e* belong to a large gene family with 10 members that all share the WD-repeat domain and show that repeats of all members of the family undergo concerted evolution. It follows that repeat units are constantly exchanged both within and between members of the gene family. As a consequence, high mutation supply in the repeat domain is ensured due to the high total copy number of repeats. We then show that in each repeat four residues located at the protein/protein interaction surface of the WD-repeat domain are under positive diversifying selection. Diversification of *het-d* and *het-e* is thus ensured by high mutation supply, followed by reshuffling of the repeats and positive selection for favourable variants. We also propose that RIP, a fungal specific hypermutation process acting specifically on repeated sequences might further enhance mutation supply. The combination of these evolutionary mechanisms constitutes an original process for generating extensive polymorphism at loci that require rapid diversification.

## Introduction

Most living organisms have developed genetic systems to discriminate self from non-self. This ability is crucial for instance for the immune response in vertebrates [1], social organisation in insects [2] or efficient out-breeding in flowering plants [3]. It has been long recognized that self/non-self discrimination is particularly critical for organisms that spontaneously form somatic chimeras, such as protists (dictyostelids and myxomycota), sponges, tunicates, ascidians and filamentous fungi [4]. In these organisms the ability to recognize and reject conspecific non-self is essential for maintenance of biological integrity. Because of their life style, these organisms are highly vulnerable to somatic cell parasitism that can even lead to germ line invasion, “the evolutionary equivalent of death” [4]. Such primitive allorecognition systems might be the evolutionary ancestor of acquired immunity in mammals as suggested by the fact that the recently isolated gene controlling somatic histocompatibility in the prochordate *Botryllus* belongs to the immunoglobulin superfamily [5]. When known, the genes involved in self/non-self recognition share a particular evolutionary signature as they appear to be under positive Darwinian selection that favours accumulation and maintenance of allelic diversity (diversifying selection) [6]. The study of the molecular evolution of this type of loci raises keen interest as their evolutionary behaviour is in sharp contrast with the vast majority of the other genes, and the mechanisms leading to high allelic polymorphism remain unclear.

In filamentous fungi, somatic cell fusion occurs spontaneously between filaments of different isolates leading to formation of a heterokaryotic structure [7] [8] [9]. In most cases however, these heterokaryotic cells are compartmentalised and destroyed by a cell death reaction. The genes controlling this somatic non-self recognition are known as heterokaryon incompatibility genes (*het* genes). Each fungal species possesses about a dozen of such incompatibility loci and a genetic difference at any one of them is sufficient to trigger destruction of the mixed cell and abortion of the fusion event. As a consequence, in most fungal species, pairs of isolates taken at random are generally incompatible. In fungi, the destruction of heterokaryotic fusion cells might also serve to limit the horizontal spreading of mycoviruses. Mycoviruses are not transmitted via the extracellular route but exclusively through cell fusion events so that incompatibility is able to limit horizontal transfer of these infectious elements [10] [11] .

A number of *het* genes have been isolated and characterized in two model species *Neurospora crassa* and *Podospora anserina* [8]. The *het-c, het-d* and *het-e* incompatibility loci of *P. anserina* are multi-allelic and define two non-allelic incompatibility systems [12]–[18] Each *het-c* allele is incompatible with a subset of *het-d* and *het-e* alleles. Interactions between incompatible *het-c/het-d* or *het-c/het-e* allele pairs lead to a cell death reaction characterized by vacuole enlargement and bursting and a simultaneous, massive induction of autophagy [19]. Autophagy is believed to prevent spreading of the cell death reaction to cells adjacent to the fusion cell [20]. *het-c* encodes a glycolipid transfer protein (GLTP) and displays a cellular function in addition to its role in allorecognition since it is required for proper ascospore formation [21]. Comparisons of different *het-c* alleles have suggested that this gene undergoes rapid evolution [16]. *het-d* and *het-e* encode large paralogous proteins that comprise three distinct functional domains. Both proteins display a N-terminal HET domain, a central NACHT domain and a C-terminal WD-repeat domain. The HET domain is a fungal-specific protein domain found in a number of proteins involved in incompatibility [22]. All fungal incompatibility gene systems-with the exception of the *P. anserina het-s/het-S* prion system-possess at least one constituent bearing a HET domain, suggesting that this domain might be involved in mediating cell death [23]. The NACHT domain is a regulatory NTP-binding domain involved in oligomerization and found in a variety of proteins controlling programmed cell death and pathogen recognition phenomena [24], [25]. Finally, the WD-repeat domain, made of a variable number of WD-40 repeat units, is a frequent repeated domain involved in protein-protein interaction. WD-repeat proteins fold into a circular β-propeller structure forming a protein-protein interaction platform. Comparison of different *het-d* and *het-e* alleles have indicated that this WD-repeat region is responsible for recognition specificity [12], [14].

In the present paper, we describe the evolutionary mechanisms responsible for diversification of the *het-d* and *het-e* allorecognition genes. We found that *het-d* and *het-e* belong to a large gene family of 10 members sharing the NACHT/WD-repeat domain organisation. We then show that the WD-repeat regions of these genesexchange repeat units and undergo concerted evolution. 4 codon positions of the WD-repeat unit are subjected to positive diversifying selection. These 4 codon positions correspond to residues forming the protein-protein interaction surface in the β-propeller structure of the WD-repeat domain. Finally, we show that two genes of the family have been inactivated by a fungal specific mutagenesis process termed RIP [26]. We propose that extensive diversification of the *het-d* and *het-e* genes is achieved by a unique combination of high mutation supply, concerted evolution and positive Darwinian selection.

## Results

### *het-D* and *het-E* belong to a large gene family

**Figure 1:**
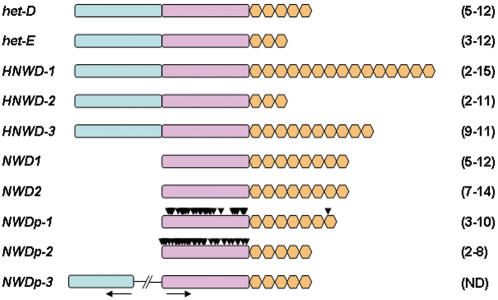
Schematic representation of the *NWD* gene family. The structural domains of the 10 members of the *NWD* gene family of the sequenced S strain are represented by blue boxes (HET domain), pink boxes (NACHT domain) and orange boxes (WD-repeat unit). Arrowheads represent stop codons in the pseudogenes. Numbers in brackets indicate the range of repeat number found in wild type isolates (Table 2). Note that *NWDp-3* pseudogene was apparently inactivated by inversion of a 60 kbp fragment splitting an ancestral *HNWD* gene in two segments with opposite orientations (arrows).

Using HET-D and HET-E protein sequences in tBlastn searches on the *P. anserina* genome (http://podospora.igmors.u-psud.fr/), we identified a number of loci encoding proteins with high sequence similarities to HET-D and HET-E. These loci were named according to their domain organisation (Table S1), and a schematic representation of the family is presented Figure 1. In addition to *het-D* and *het-E*, three more loci comprise genes encoding for the same three domain proteins. They will be grouped under the generic name HNWD genes. We also identified two genes encoding for proteins associating only the NACHT and the WD-repeat domains, named *NWD1* and *NWD2*. Three pseudogene copies of *NWD* genes were also found. Two of them (*NWDp1* and *NWDp2*) contain numerous non sense mutations, whilst the third pseudogene (*NWDp3*) is lacking a start codon. *NWD3p* is immediately preceded by the relic of a transposable element. 60 kbp upstream, a gene encoding for a single stand alone HET domain, transcribed in the opposite direction to *NWDp3*, is present. It is conceivable that these two sequences constitute the remains of an ancestral *HNWD* gene that was split in two by the inversion of a this 60 kbp fragment. We also found 120 additional loci encoding proteins displaying a HET domain. These loci will not be analyzed further here, but note that the presence of multiple loci encoding HET proteins was already described in a number of fungal species, including *N. crassa* and *Aspergillus fumigatus* [27], [28].

We conclude that *het-D* and *het-E* belong to a large gene family containing both active and pseudogene members that share NACHT and WD domains.

### *NWDp1* and *NWDp2* pseudogenes have been inactivated by RIP

Repeat Induced Point Mutation (RIP) is a process first described in *N. crassa* that detects and mutates both copies of a duplicated sequence. Providing that the nucleotide sequences are over 80% identical, RIP is active on linked duplicated copies of a sequence over 400 bp in length, or over 1 kbp if the copies are unlinked. RIP is thought to have evolved as a defence mechanism against transposable elements. Evidence of RIP have been collected from a number of species, including *P. anserina* [29]. RIP incorporates C:G to T:A mutations, preferentially in CpA dinucleotides [26], [30]. RIP reduces GC content of mutated sequences and results in skewed CA/TA and TG/TA ratios.

The two pseudogenes *NWDp1* and *NWDp*2 are heavily mutated compared to wild type *NWD1* sequence. To assess the hypothesis that *NWDp1* and *NWDp2* pseudogenes are RIP inactivated, we first determined the GC content of NWD family members. As reported in Table S2, GC content of pseudogenes *NWDp1* and *NWDp2* is significantly lower than for other family members. We then measured CA/TA and TG/TA ratios at all the loci of interest in the regions encoding the three functional HET, NACHT and WD domains (Table 1). Also, as RIP is known to be a leaky process that introduces mutations in and around the target duplicated sequences, we also estimated the CA/TA ratio in the regions surrounding the genes of interest. Reference values (CA/TA = 2.244 and TG/TA = 2.225) were obtained by analysing 20 randomly chosen 750 bp long genomic sequences. CA/TA and TG/TA ratios for the NACHT domain of the pseudogenes *NWDp1* and *NWDp2* are significantly skewed compared to NACHT domains of other family members. We also observe that low CA/TA and TG/TA ratios extend at least 750 bp upstream and downstream of the pseudogenes. From the above observations, we conclude that pseudogenes *NWDp1* and *NWDp2* have been inactivated through the RIP process. Note that considering the relatively low efficiency of RIP in *P. anserina* [29], RIP levels in *NWDp1* and *NWDp2* are very high. For example no CpA dinucleotides are found in the 750 bp upstream of the *NWDp2* pseudogene, compared to 44 CpA dinucleotides found in the corresponding region of functional *NWD1*, strongly suggesting that multiple rounds of RIP acted on the pseudogenes.

**Table 1:** CA/TA and TG/TA ratios at *NWD* loci.

	<−2250 −1500>	<−1500−750>		<−750 0>		HET		NACHT		WD		<0+750>		<+750+1500>		<+1500+2250>	
	CA/TA	TG/TA	CA/TA	TG/TA	CA/TA	TG/TA	CA/TA	TG/TA	CA/TA	TG/TA	CA/TA	TG/TA	CA/TA	TG/TA	CA/TA	TG/TA	CA/TA	TG/TA
*NWDp-1*	0.76	1.03	0.64	**0.21**	**0.13**	**0.006**	-	-	**0.1**	**0.12**	3.28	1.55	**0.19**	**0.21**	**0.5**	0.63	1.31	1.21
*NWDp-2*	0.88	1.08	**0.09**	**0.09**	**0**	**0.02**	-	-	**0.03**	**0.01**	3.24	1.56	**0.002**	**0.07**	0.78	0.72	2.9	2.24
*NWDp-3*	0.87	1.05	0.98	1.17	1.64	1.17	-	-	1.15	1.08	**0.74**	0.42	0.85	0.81	1.05	0.57	3.12	3.12
*NWD1*	2.47	4.4	3.2	2.28	2.52	2.52	-	-	2.01	1.74	3.35	4.9	1.04	1.21	1.69	2.11	2.47	1.69
*NWD2*	1.67	1.24	1.8	1.61	2.67	2.05	-	-	1.92	1.32	1.59	1.22	0.89	0.93	2.62	2.86	1.86	1.32
*het-D*	1.39	0.81	1.31	0.91	1.93	1.66	1.25	1.16	1.13	1.16	2.06	1.59	0.77	0.87	1.66	2.12	1.17	1.66
*het-E*	2.22	1.93	0.78	1.16	1.04	0.84	1.89	1.92	1.43	1.29	1.15	0.83	1.02	1.05	1.97	1.08	1.26	1.44
*HNWD1*	2.37	1.74	0.91	1.29	1.48	1.40	1.96	1.65	1.12	0.96	1.65	1.12	0.91	0.66	**0.46**	**0.17**	**0.11**	**0.08**
*HNWD2*	0.82	1.13	1.8	2.07	1.47	1.5	1.54	1.69	1.2	1.04	1.51	1.4	1.36	1.02	0.83	1.09	0.7	1.06
*HNWD3*	**0.39**	**0.24**	0.98	0.56	1.75	0.89	2.09	1.86	1.55	1.35	2.38	0.96	0.51	0.57	1.5	2	1.86	2.21
Lower Value CI 99%	0.606	0.302	0.368	0.387	0.556	0.377	1.038	1.04	0.484	0.449	1.132	0.287	0.332	0.361	0.586	0.433	0.692	0.766

*NWD* loci were sliced as the three functional domain encoding sequences, and successive 750bp regions upstream and downstream of the genes were analysed. Significantly lower values at a 99% confidence interval are bold. As a control, CA/TA and TG/TA ratios for 20 different randomly selected 750bp regions of *P. anserina* genome were 2.244+/− 0.854 and 2.225+/− 0.992 respectively. Low ratios downstream of *HNWD1* appear to result from RIP of a repeated sequence related to a *Tad3-2* LINE element and sequences upstream of HNWD3 match *P. anserina* centromeric DNA.

Intriguingly, the CA/TA and TG/TA ratios in the WD domain of *NWDp1* and *NWDp2* loci are comparable to those observed in the WD domains of all the active loci, and to that of the rest of the genome. Translation of the NACHT domains of the pseudogenes reveals an accumulation of non sense mutations. In contrast, the WD domain of the *NWDp2* locus is devoid of stop codons over a total length of 5 repeats of the WD40 unit, whilst in the *NWDp1* WD domain, a single stop codon occurs at the beginning of the 7^th^ of the WD-40 unit (Figure 1).

In conclusion, the *NWDp1* and *NWDp2* pseudogenes have been subjected to extensive RIP. However, this high level of RIP is not observed in the WD-40 sequences of the pseudogenes. This observation might be explained by hypothesizing that WD-repeats of the pseudogenes undergo concerted evolution with the WD repeats of the active loci so that repeats in the pseudogenes can be replaced by repeats originating from the active loci.

### The WD-40 repeats undergo concerted evolution both within and between loci

In addition to the presence of intact WD-40 repeats in the pseudogenes loci outlined above, a second observation suggested concerted evolution of the WD repeats of the *NWD* gene family. Individual WD-40 repeats of the gene family are extremely conserved within a gene as well as between genes with up to 100% identity. This conservation level is striking considering that the WD-40 motif is loosely defined with only a few key residues critical for the β-propeller fold. In other WD-repeat proteins in *P. anserina* and other species, conservation of repeat units rarely exceeds 10 to 20% at the protein level [31] and is in most cases barely detectable at the nucleotide level. Concerted evolution is a process that leads to homogenization of repeated sequences. Concerted evolution is driven by unequal crossing overs and/or gene conversion events in repeat arrays. During concerted evolution a mutation in any given repeat can ultimately either spread to all of the repeats or be eliminated, as a result all the repeats evolve as a unit. Concerted evolution is detected in phylogenetic analyses by sequences grouping as species of origin rather than as orthologues [32].

**Figure 2:**
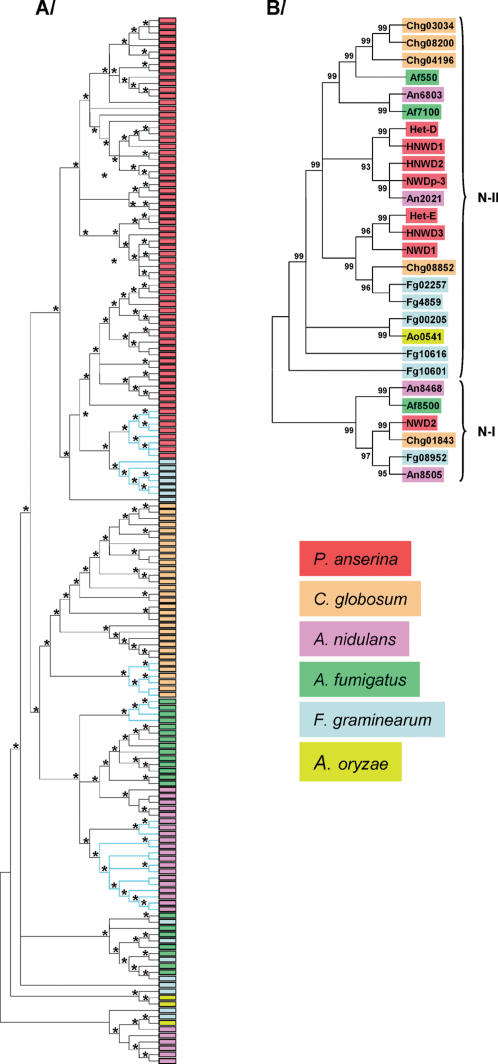
Concerted evolution of the WD-40 repeats. A/Schematic representation of a Neighbor-Joining phylogenetic tree of individual WD-40 sequences. Each box represents a single WD-40 sequence. Asterisks indicate Internal Branch Length tast values over 80. The corresponding full size tree is presented on figure S3. B/Neighbor-Joining phylogenetic tree of NACHT domain sequences. Each WD-40 unit is designated by its gene of origin and position in the domain. Cyan branches indicate sequences associated to NACHT domains belonging to N-I clade of the NACHT phylogeny. Internal Branch Length test values over 80 are indicated. For both trees, species of origin are colour coded.

To perform this type of phylogenetic analysis we searched for sequences similar to HET-D and HET-E in the fungal databases. Interestingly, *HNWD* type of sequences were not found in any other organism, even in closely related species such as *N. crassa* or *Chaetomium globosum*. However, *NWD* orthologues were identified in the genomes of 5 additional species, *C. globosum, Giberella zeae, Aspergillus nidulans, Aspergillus oryzae* and *Aspergillus fumigatus*. An ITS based phylogenetic tree of these species is presented on Figure S1. All available *NWD* sequences were collected (Table S3) and a phylogenetic tree of all the individual WD40 units was constructed using Kimura two parameter distances. We conducted bootstrap and interior branch tests available in the MEGA 3.1 package to assess statistical significance of the phylogenetic trees. Both tests resulted in different statistical support of the tree produced as already reported [33]. In this WD-repeat phylogenetic tree, all *P. anserina* WD-40 repeat sequences cluster together, including repeats from the pseudogenes (Figure 2A and S2). The *P. anserina* clade also includes WD-40 sequences from a single *F. graminearum* gene, possibly revealing a common ancestral origin. Similarly, all WD40 sequences from *Ch. globosum* group together. Other WD-40 sequences are mixed up. The clustering of *P. anserina* sequences was well supported by the interior branch test, but only the two main internal nodes were well supported by bootstrapping analysis. Note that interior branch test is more reliable for closely related sequences [33]. Another striking point is that even if WD-40 sequences from *het-D* and *HNWD1*, and from *het-E* and *HNWD3* are mixed (Figure S3), WD-40 sequences tend to cluster by gene of origin, suggesting preferential intralocus recombinations. Note that parsimony trees lead to the same conclusions (Figure S4)

“Whole-gene” and NACHT phylogenetic trees were also constructed with the same genes. In the NACHT domain phylogeny the RIP inactivated NACHT domains were excluded because they are too divergent for proper alignment. The “whole-gene” and NACHT phylogenetic trees resulted in similar topologies which was to be expected as the NACHT domains are more divergent than the WD-repeat domains and hence outweight the influence of the WD-repeat on the tree topology. Both phylogenies gave evidence for two well supported main clades (N-I and N-II, Figure 2b). The smallest clade N-I contains *P. anserina* *NWD2*, as well as at least one representative from all the other species. N-II includes the remaining sequences. Within N-II, *P. anserina* sequences are spread over two well supported sub-clades each also containing sequences from other species. The statistical support by the bootstrap or the internal branch method for both the “whole-gene” phylogeny and the NACHT phylogeny are roughly in agreement.

The fact that all *Ch. globosum* and *P. anserina* WD-40 sequences group as species rather than orthologs is proof of concerted evolution of the WD-40 sequences. An alternative hypothesis to concerted evolution of the WD40 sequences would be a recent duplication of the genes in Podospora or a closely related ancestor. However, the grouping of WD-40 sequences from the N-I and N-II NACHT domain clades allows to reject this hypothesis. Additional data support the hypothesis of concerted evolution. The presence in *P. anserina* of functional WD domains at the pseudogene loci is readily accounted for by concerted evolution of the repeats but not in the recent gene expansion hypothesis. From the above observations, we conclude that the WD-40 sequences from *P. anserina* (and incidently *Ch. globosum*) are undergoing concerted evolution. In other words, the repeat units of the entire gene family undergo genetic exchanges by unequal crossing-over and/or gene conversion and as a result they evolve in concert. These phylogenetic analyses indicate that potentially each repeat unit at any given locus from the gene family can be recombined with any repeat of any locus of the family. It follows that any given repeat is a potential target for mutations that can then be introduced in repeat arrays of other family members. As a consequence, mutation supply in the repeat region is significantly increased. The total number of templates for mutation is 69 repeats in the sequenced *S* strain.

### WD repeat number is highly variable in all members of the family

If driven by unequal crossing-overs, concerted evolution will result in repeat array size polymorphism. Repeat number polymorphism in wild *P. anserina* isolates was already reported for *het-D* and *het-E* alleles [12]. We determined the number of WD-40 repeats by PCR amplification of the WD-repeat domain from each *NWD* locus in wild *P. anserina* isolates. We observed an extensive repeat number polymorphism (Table 2). No two isolates have the same WD-40 unit number distribution pattern suggesting unequal crossing-overs contribute to WD-40 sequence redistribution. Note however that the total number of WD repeats is constantly high, with over 50 repeats of the WD-40 unit in each isolate.

In agreement with the detection of concerted evolution in the WD-40 repeats, we show that all members of the gene family show extensive repeat number polymorphism suggesting that repeats are actively exchanging in wild populations.

**Table 2:** WD-repeat number polymorphism.

	Wild P. anserina isolates						
Loci	D	E	M	Cs	S	Y	Z
*het-D*	11	7	12	12	5	11	11
*het-E*	12	10	10	3	3	10	ND
*HNWD1*	2	2	13	13	15	13	13
*HNWD2*	4	4	11	2	3	4	4
*HNWD3*	10	10	9	10	10	11	10
*NWD1*	5	10	10	12	>8	9	9
*NWD2*	7	10	10	7	8	12	14
*NWDp-1*	ND	ND	3	9	7	ND	10
*NWDp-2*	ND	2	8	7	5	8	4
*NWDp-3*	ND	ND	ND	ND	5	ND	ND
Total WD-40	>51	>55	>86	>75	>69	>78	>75

WD-40 repeat numbers of WD domains of the *NWD* gene family members in *P. anserina* wild isolates as determined by PCR analysis. Data for the isolate S are from the genome sequence. ND, not determined. Note that especially in the pseudogenes, PCR amplification failed probably because of RIP-induced polymorphism.

### 4 codon positions of the WD-40 repeats are under positive diversifying selection

Positive Darwinian selection is common in self/non self recognition genes [6] and describes the fact that acquisition of non synonymous mutations is favoured hence promoting evolution of the encoded protein [32], [34], [35]. Since the WD-repeats control recognition specificity, we assessed the possibility that they might be under positive Darwinian selection. The most common test for detecting direction of selection is to determine the Dn/Ds ratio, i. e. comparing the ratio of non synonymous mutation per non synonymous site to the ratio of synonymous mutations per synonymous sites. A Dn/Ds ratio close to 1 is indicative of neutral evolution. Dn/Ds<1 reveals purifying selection whereas Dn/Ds>1 indicates positive selection.

**Figure 3:**
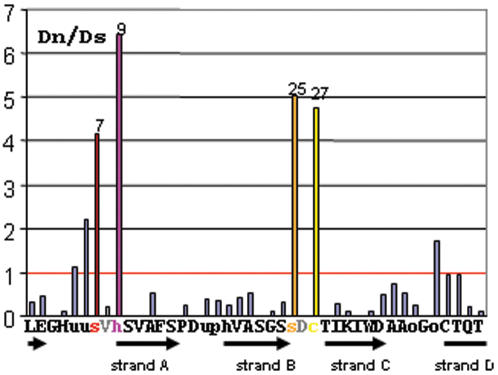
Positive selection on the WD-40 repeat sequences. Dn/Ds ratio calculated by the FEL method (p-value<0.05) represented along a consensus of the WD-40 amino acid sequence (u = A, G or S, o = S or T). Black arrows indicate regions of the WD-40 sequence forming β-strands.

Since the WD-40 of the family evolve as a unit, in concert, their comparison is evolutionary meaningful. We performed two different types of analysis using the whole set of 69 *P. anserina* WD40 sequences from the sequenced *S* strain, the Suzuki and Nei counting model (1999), and the Fixed Effect Likelyhood (FEL) implemented by HyPhy [36]. Both models identified 4 codons (codons number 7, 9 25 and 27) under strong positive selection, and a number of less strongly selected positions (Figure 3). Sites under strong positive selection work in pairs, 2 positively selected codons surrounding a site under strong purifying selection (codons 8 and 26). Purifying selection at position 8 and 26 is fully consistent with the fact that these positions are part of the WD-repeat consensus [31] .

Together the 69 WD-40 repeat units present in the genome of the sequenced *S* strain form 55 different combinations of amino acids at the 4 positions under positive selection (Table S4). This observation suggests that the WD-40 sequences are evolving under diversifying selection rather than adaptive-directional-selection [34].

We conclude that selection promotes acquisition and maintenance of a high number of polymorphism at these specific positions in the pool of WD-40 sequences of the *NWD* gene family.

### The residues under positive diversifying selection in the WD-40 repeats group at the protein-protein interaction platform

**Figure 4:**
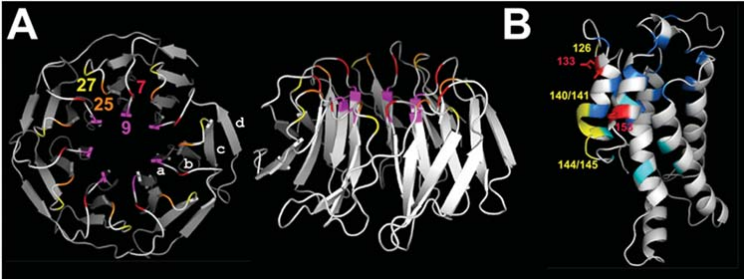
Analogy based modelling of interacting partners. A/Top and side view of the predicted structure of the HET-E WD repeat domain. Positions under strong positive selection are coloured. B/Predicted structure of the *het-C* gene product encoding a glycolipid transfer protein. Red amino acids correspond to positions experimentally shown to be involved in recognition specificities, yellow amino acids represent highly polymorphic positions between *het-C* alleles. Note that residue 126 is located in a unstructured loop, so its positioning is uncertain. Residues forming the sugar binding site are coloured in cyan, residues forming the lipid binding tunnel are coloured in blue. Polymorphic residues are located outside the sugar binding site and the lipid binding hydrophobic tunnel.

The WD repeats adopt a circular structure with a central pore called the β-propeller that assembles 7–8 WD units, each repeat being composed of 4 short antiparallel β-strands (*a, b, c* and *d, a* being the closest to the central pore). In each subdomain, strand *d* from one repeat associates with strands *a, b* and *c* from the next repeat. The β-propeller displays a flat rigid protein-protein interaction platform at the top of the structure. Surface residues of this platform are responsible for protein/protein interactions in WD proteins such as Tup1 [37], the Gβ subunits [38] and net2 [39] (For a review [31]). A structural model of the WD-40 repeat domain of HET-E was generated by homology modelling to WD-40 domains of known structure. When positioned on this homology-based three dimensional model of the HET-E WD domain, the residues corresponding to the 4 codon positions under positive selection are all located on the top of the β-propeller on the surface of the interaction platform (fig. 4a). Pair (7, 9) is located in the loop connecting strand *d* from one repeat to strand *a* in the next repeat for and pair (25, 27) in a β-turn linking strand *b* and *c* within a repeat (fig. 4a). This positioning in the WD domain of the residues under selection strongly suggests that they are involved in protein-protein interactions.

*het-C* is the interacting partner of *het-D* and *het-E* in incompatibility. Incompatibility is triggered by interaction of specific allele pairs of *het-C* and *het-D*, and *het-C* and *het-E*. *het-C* encodes for a glycolipid transfer protein that also assumes developmental functions [21]. Although the restricted number of *het-C* allele sequences prevents genesis of statistically significant results, comparison of the four known alleles led to two important observations. First, *het-C* alleles are more variable in the open reading frame than in 5′ or 3′ non coding regions and introns with about twice as many mutations in coding regions as in non coding regions. Second, polymorphism in the open reading frame results in an excess of non synonymous over synonymous mutations, with only two synonymous mutations observed for 15 non synonymous mutations between the two most divergent alleles [16]. These observations are suggestive of positive selection acting on *het-C*. At least 2 codons in positions 133 and 153 involved in determining allelic specificities with *het-D* and *het-E* were identified, as well as a highly polymorphic region comprising codons 126, 140/141 and 144/145 [16]. As for the HET-E WD repeat domain, we generated a structure model of HET-C by homology modelling to human GLTP [40]. On this structure model, residues 126, 133, 153, 140/141 and 144/145 are all located at the surface of the HET-C protein roughly in the same region, and are not part of the hydrophobic tunnel nor the sugar binding site involved in glycolipid binding (Figure 4B) [40], [41]. These data suggest that variable positions in HET-C could form an interaction interface with WD domains of HET-D and HET-E, and potentially that the WD sequences and *het-C* are co-evolving.

## Discussion

### Genesis of new allorecognition alleles

Most living organisms, from bacteria to higher eukaryotes, have developed allorecognition systems to discriminate self from non self tissues and cells. A remarkable property of recognition genes is their ability to accumulate polymorphism, they are often under positive Darwinian selection [34]. It is generally accepted that balanced selection that necessarily involves some type of rare allele advantage contributes to fixation of rare advantageous alleles [6]. However, although highly polymorphic sequences are central for allorecognition, the mechanisms of their genesis are often unclear. The low mutation rate in most cellular organisms imposes a serious limitation on diversification and adaptation in general [42], and particularly so in small or well adapted populations [43]. In fungi, allorecognition functions are ensured by vegetative incompatibility loci.We show here that the combination of high WD-40 repeat sequence number, concerted evolution and positive selection provides the *P. anserina NWD* gene family-which includes several allorecognition genes-with a dynamic system by which mutations in WD40 repeats can be acquired and spread to the whole family and positively selected if advantageous. All the 70 or so WD-40 units of the *NWD* family members, the basic elements of the WD recognition domain, are multiple potential targets for mutagenesis which *per se* might already be a significant enhancement of mutation supply. The fact that RIP, as a repeat-induced hypermutation process might also contribute to increase mutation supply and diversification of the WD-40 repertoire is discussed in a following section. Concerted evolution will then allow redistribution of repeat variants from this large WD-40 sequence repertoire between family members. Finally and importantly, positive selection will filter mutations in the loops of the interaction platform of the β-propeller structure to retain favourable mutations. In addition, the concerted evolution process will also ensure constant homogenization of the remaining of the WD-40 sequence thereby maintaining competency for further rounds of redistribution.

### To what extent RIP contributes to WD-repeat sequence diversification?

Two pseudogene members of the NWD family have been inactivated by the fungal specific hypermutation process called RIP. RIP introduces CT to TA and TG to TA mutations, increasing chances of introducing amber and ochre stop codons. RIP was first described as a defence mechanism against transposable elements. Indeed, relics of transposable elements riddled with stop codons have been found in a number of species including *P. anserina* [44], *Leptospharia maculans* [45] and Aspergillus species [46]. In *N. crassa*, RIP mutated sequences are methylated, resulting in their transcriptional silencing [26]. However, despite thorough investigation, DNA methylation could not be associated to RIP in Aspergillus species [46], and to the best of our knowledge RIP associated methylation has never been reported for any organism other than *N. crassa*. Also, RIP mutagenesis is by far the most efficient in *N. crassa*. Consequently, one function of low efficiency RIP might be to promote evolution of repeat sequences [26], [47], while high mutagenesis and transcriptional silencing add extra strength to genome defence in *N. crassa*.

When excluding the WD repeat sequences, RIP inactivated *NWDp1* and *NWDp2* pseudogene sequences are about 80% identical, corresponding to the threshold level beyond which RIP is believed to become ineffective [26]. *NWDp1* and *NWDp2* are most likely the products of a gene duplication, both duplicated copies being subsequently inactivated by multiple rounds of RIP. This inactivation must have introduced RIP mutations within the WD-repeat sequences as well, thereby contributing to WD sequence diversification. It can thus be stated that at least at the time of the initial RIPping of the *NWDp1* and *NWDp2* genes, RIP has provided a mutation input in the WD-40 repeat repertoire. Sequences over 80% identical and over 1 kbp long are found in WD-repeat arrays of *P. anserina NWD* gene family, thus-if these repeats are no refractory to RIP for some unknown reason-, the WD-repeats should *a priori* trigger RIP. We have counted the number of RIP characteristic mutations (C to T and G to A transitions in a CA or TG context) at silent positions only (third base of each codon) to avoid any effect of selection. The sample of such potential selection-free RIP targets is limited and prevents convincing statistical analysis, however as expected under RIP, we noted that most CpA dinucleotides (8/9) are mutated, and number of C to T transitions are more frequent at CpA dinucleotides (78%) as compared to other dinucleotides (around 40%) (Table S5). These observations are compatible with the hypothesis that RIP might be occurring in WD-repeat sequences, so that these repeats might be constantly promoting their own diversification. Clear footprints of RIP might well be blurred by the purifying and positive selection acting on the repeats as well as the concerted evolution process which *per se* promotes homogenisation. Only experimental determination of the levels of RIP in the WD-repeats in the absence of selection will allow to determine to what extent RIP participates to mutation supply in this gene family. The two extreme possibilities are that RIP introduced mutations in the repeat pool only at the time of duplication of *NWD1p* and *NWDp2* or that WD-repeat triggered RIP constantly allows locus specific hypermutation of the repeat arrays.

### Positive selection on *het* genes

Acquisition/maintenance of new allele specificities at *het* loci in different fungal species rely on extremely unusual processes. In *O. novo-ulmi*, the dutch elm disease pathogen, acquisition of new *het* alleles (as well as mating type alleles) occurs through introgression from a closely related species [48]. In *N. crassa*, balancing selection is acting at the *het-C* locus to maintain three functional alleles, and evidences for ancient trans-species polymorphism were presented [49]. These observations highlight the essential role that vegetative incompatibility must play in fungal biology. This conclusion is now comforted by the fact that we identified positive Darwinian selection acting on positions forming the protein/protein interface of a recognition gene family. Note that genes controlling programmed cell death seem to be a target of choice for selection to act on. A*paf-1* encoded protein controlling apoptosis in mammals display a structural organisation similar to proteins of the *HNWD* gene family with a N-terminal CARD effector domain, a central NACHT domain and a C-terminal domain containing 12 WD-repeats Recently, it was shown that *apaf-1*, is also subjected to positive selection acting on the WD domain [50].

Among the NWD genes, only *het-D* and *het-E* have been characterized as vegetative incompatibility genes so far [12]. It is likely that other members of the family also correspond to incompatibility genes, in particular those displaying a HET domain characteristic of proteins related to incompatibility [12], [15], [23], [51], [52]. Molecular genetic analyses indicate that *HNWD2* in either tightly linked or congruent to the *het-R* incompatibility gene [53], (D. Chevanne and M.P., unpublished results).

Clearly positive diversifying selection is acting on the WD-repeats of this set of genes, but what is actually selected? Combining the large number of different WD-repeats to the ability to re-shuffle them by concerted evolution has the potential to generate very large numbers of non self recognition alleles. However, an incompatibility system is defined by the interaction of two partners and in the case of *het-d* and *het-e,* this partner is the *het-c* gene. Four alleles of *het-C* have been identified in a sample of 16 wild isolates, which is not in terms with the level of variability in the WD-40 sequences. This leads to an apparent paradox : what is the use of being able to generate very large numbers of *het-d* and *het-e* alleles when there are only 4 *het-c* alleles around. Several hypotheses can be envisaged to explain this discrepancy. Using chimeric *het-C* alleles it was shown that new interaction specificities can be obtained experimentally [16]. So, one may hypothesize that other yet uncharacterized natural *het-C* alleles exist in wild populations. Alternatively, *het-C* may not be free to diverge extensively because it assumes a cellular function in spore formation [21]. Thus, it might be the number of *het-C* alleles is indeed limited to the four known alleles. Then how can one explain the diversity in *het-D* and *het-E*? As stated above, the other *HNWD* genes are likely additional incompatibility genes whose interacting partners are unknown, so that incompatible interactions with *het-c* may not be the only interactions that promote divergence of the repeats. Indeed the *het-R*/*het-V* system is independent of *het-C* (unpublished).

An altogether different view would be that selection is not acting on emergence of novel phenotypic classes but on repeated emergence of incompatibility towards the same four *het-c* alleles. Mutation and re-shuffling of the WD-40 repeats would perpetually restore already existing phenotypes. One striking characteristic of the incompatibility system we describe here is its dynamic character, in contrast to incompatibility systems such as the *N. crassa het-C* system that has maintained the same three alleles in different species over tens of million years of evolution (Wu et al 1998). As a consequence of this dynamic character, *het-e* and *het-d* alleles seem to be genetically unstable. In spite of the strong positive selection in the repeats-which strongly argues for an adaptive advantage associated to incompatibility-numerous wild isolates have inactive *het-d* and *het-e* alleles as a result of repeat loss, as for instance in the sequenced *S* strain. Similarly, in genetic screens selecting for loss of incompatibility, *het-d* and *het-e* inactivation through WD-repeat loss is by far the most frequent event [54]. As a flipside of the dynamic character of the concerted evolution process, the *het-d* and *het-e* incompatibility genes appear to be genetically fragile. It appears however that in every analysed wild isolate at least two *HNWD* genes contain a minimum of 10 repeats, the apparent lower limit for activity in incompatibility [14], [15]. It might be that the signature of positive selection in the WD-repeats results from reoccurring episodes of loss and subsequent reacquisition of the incompatibility function.

### General implications

Fast evolving sets of genes have been found in various organisms. For instance, immunoglobulin genes in mammals evolved through a combination of high gene copy number, DNA shuffling between exons of the genes into expressed sites, and high mutation rate to optimise recognition (for a review, [55]. In parasites such as *P. falciparum*, antigen surface encoding genes need to evolve rapidly to avoid recognition by the immune system, and this is achieved by DNA shuffling between a single expressed site and numerous silent loci, introducing mutations in the process [56]. In plants, production of a variety of resistance genes to pathogens is promoted through shuffling of sets of resistance genes [57], and in the fungus *L. maculans*, RIP could promote evolution of an *avr* gene located in a gene desert heavily subjected to RIP [45]. In bacteria, high mutation rate associated to mutator genes allow rapid adaptation to new selective conditions [58]. In *P. anserina* genesis of new allorecognition specificities relies on the combination of the DNA shuffling of a large repertoire of repeats between expressed and non expressed loci and a step of selection for advantageous new variants. RIP as a repeat specific hypermutation process could further contribute significantly to diversification. It would then seem that organisms have evolved or exploited various mechanisms to achieve rapid gene diversification through strategically similar processes involving high mutation input, DNA sequence re-shuffling and positive selection.

## Materials and Methods

### Sequence mining

Sequences were identified by tblastn searching of the *P. anserina* database (http://podospora.igmors.u-psud.fr/), the NCBI database (http://www.ncbi.nlm.nih.gov/), the Broad Institute Fungal Genome Initiative database (http://www.broad.mit.edu/cgi-bin/annotation/fgi/blast_page.cgi), the TIGR *Aspergillus fumigatus* database (http://www.tigr.org/tdb/e2k1/afu1/), and the *A. oryzae* database (http://www.bio.nite.go.jp/dogan/Top) with *het-D* and *het-E* sequences. NACHT and WD-repeat domains from non annotated sequences were identified using the Prosite program (http://www.expasy.org/prosite/). For *P. anserina* genes, HET domain [22] corresponds to the extension N-terminal to the NACHT domain. All sequences are presented in Tables S1 and S3.

### Positive selection analysis

For positive selection detection, the Fixed Effect Likelihood model (FEL) implemented in HyPhy [36], [59] was used with the HKY85+G substitution model found to be the best matrix by HyPhy's model selection program. We also ran a SLAC method derived from the Suzuki and Gojobori model [60], implemented as a Web interface on the www.datamonkey.org web site. Consensus protein sequence of the WD-40 unit was determined by the Consensus program (http://coot.embl.de/Alignment/consensus.html). Cumulative behaviour of the *het-C* alleles was determined using the program SNAP implemented as a web site interface (http://www.hiv.lanl.gov/content/hiv-db/SNAP/WEBSNAP/SNAP.html) based on Nei and Gojobori method [61].

### Phylogenetic analysis

Sequence alignments were performed with ClustalW, and phylogenetic analysis were computed using the MEGA3.1 package [62]. Neighbor-Joining trees [63] were constructed based on Kimura 2-parameters distances [64]. Internal Branch Tests were conducted using 1000 replicates. Parsimony phylogenies were also determined and bootstrap analysis conducted with 1000 replicates. RIP inactivated NACHT sequences as well as *A. oryzae* WD sequences from locus Ao0041 were excluded because too divergent for proper alignment. Neighbor-joining is a distance based method of phylogeny reconstruction. In the Parsimony approach, for any given topology the sum of the minimum possible substitutions over all sites is known as the tree length for that topology. The topology with the minimum tree length is known as the Maximum Parsimony tree. Merits and demerits of these methods were discussed [33].

Highly conserved gene families have been shown to evolve according to either of two processes, “Birth and Death with Strong purifying Selection” or “concerted evolution” [32], [35]. In the Birth and Death with purifying selection model duplicated sequences evolve separately in a genome, and purifying selection will ensure conservation of the sequences. In this case, genes of a family will cluster as orthologues. In contrast, in the concerted evolution process, members of a family evolve as a unit within a genome by unequal crossing-overs and/or gene conversion and sequences will then group as species of origin rather than as orthologues.

### Structure modelling

HET-E1 and HET-C2 structure models were generated by homology modelling using the Geno3D web interface (http://geno3d-pbil.ibcp.fr/) [65], graphic representations were made with Pymol.

### PCR

PCR amplifications were performed with 10 ng genomic DNA, 100 ng of each primer, 100 µM each dNTP, 1×Taq polymerase buffer containing magnesium chloride, 1U Taq DNA polymerase (Qbiogen). After 5 min denaturation at 95°C, 35 cycles of 30 sec at 95°C, 30 sec at 55°C and 3 min at 72°C were performed before gel electrophoresis. Primers are listed on Table S6.

## Supporting Information

Figure S1 Phylogenetic relationship of the species under study. An ITS sequence Neighbor-Joining phylogeny was constructed and bootstrap tests conducted with 1000 replicates (bold). Genetic distances are indicated. Accession numbers for the ITS sequences are: AY278557 (P. anserina), DQ336707 (C. globosum), DQ453701 (F. graminearum), AJ937756 (A. nidulans), DQ401534 (A. fumigatus) and DQ411551 (A. oryzae).
(0.01 MB PDF)

Figure S2 Close up view of the P. anserina clade presented Fig. 2B. WD-40 repeats are designated by the gene of origin and their position from the first WD-40 repeat at the N-terminal end of the WD-repeat domain. Genes of origin are colour coded. Cyan branches indicate sequences associated to NACHT domains grouping in the N-I clade of the NACHT phylogeny. Internal Branch Length test values over 80 are indicated.
(0.02 MB PDF)

Figure S3 Full size WD-40 unit Neighbor-Joining phylogenetic tree identical to figure 2A. Loci of origin are noted, and species of origin are colour coded. Each WD-40 unit is designated by the gene of origin and the number of the WD-40 repeat from the N-terminal end of the domain. Cyan branches indicate sequences associated to NACHT domains grouping in the N-I clade of the NACHT phylogeny. Internal Branch Length test values over 80 are indicated.
(0.02 MB PDF)

Figure S4 Maximum Parsimony phylogenetic trees constructed with the same data set as Neighbor-Joining tree reported Fig. 2. A/NACHT phylogeny, B/WD-40 phylogeny. Loci of origin are noted, and species of origin are colour coded. Each WD-40 unit is designated by the gene of origin and the number of the WD-40 repeat from the N-terminal end of the domain. Cyan branches of the WD-40 phylogeny indicate sequences associated to NACHT domains grouping in the N-I clade of the NACHT phylogeny. Bootstrap values over 50 are indicated.
(0.03 MB PDF)

Table S1 Accession numbers of P. anserina NWD family genes Genes newly identified were named according to their structural composition. H = HET domain, N = NACHT domain, WD = WD-repeat domain. Pseudogenes are indicated by the suffixe p.
(0.02 MB PDF)

Table S2 GC content of the NWD family GC content of the NWD loci were determined for each member of the gene family. Control values (CTL) were defined as the GC and TA contents averaged over twenty randomly chosen 750bp control sequences. Shaded values are significantly lower than the rest of the dataset at a 95% confidence interval.
(0.02 MB PDF)

Table S3 Genes of the NWD family identified in other fungal species.
(0.02 MB PDF)

Table S4 55 combinations of amino acids at the positions under positive selection in the WD-40 repeats of the P. anserina NWD gene family. WD sequences are named as in figure 2. Identical combinations are colour shaded, and the number of different amino acids at each position is indicated
(0.05 MB PDF)

Table S5 Mutation frequencies at silent positions in the WD-40 sequences. WD-40 sequences were analysed as two pools according to their belonging to one of the two main clades in the WD40-phylogeny presented figure 2. One pool comprised WD-40 sequences from loci NWD-1, het-E, NWDp1, NWDp2, NWDp3 and HNWD3, the other pool comprised sequences from loci NWD2, Het-D, HNWD1 and HNWD2. C to T transitions in the pool of WD-40 sequences compared to the consensus sequences were counted on both strands and are reported according to their context.
(0.02 MB PDF)

Table S6 Primers used to PCR amplify WD repeat domains from P. anserina NWD gene family members.
(0.02 MB PDF)
