# Supplementary material for: Genesis of a Fungal Non-Self Recognition Repertoire
Source: PLoS One. 2007 Mar 14;2(3):e283. doi: 10.1371/journal.pone.0000283 (PMC1805685; doi:10.1371/journal.pone.0000283)
Supplement: Figure S1 — Phylogenetic relationship of the species under study. An ITS sequence Neighbor-Joining phylogeny was constructed and bootstrap tests conducted with 1000 replicates (bold). Genetic distances are indicated. Accession numbers for the ITS sequences are: AY278557 (P. anserina), DQ336707 (C. globosum), DQ453701 (F. graminearum), AJ937756 (A. nidulans), DQ401534 (A. fumigatus) and DQ411551 (A. oryzae). (0.01 MB PDF) [file pone.0000283.s001.pdf]

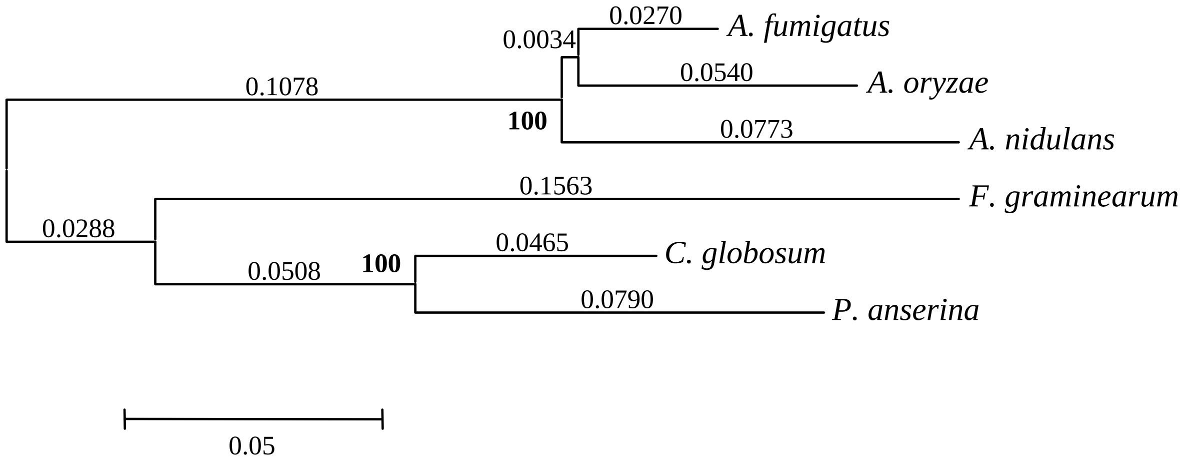

Figure S1: Phylogenetic relationship of the species under study. An ITS sequence Neighbor-Joining phylogeny was constructed and bootstrap tests conducted with 1000 replicates (bold). Genetic distances are indicated. Accession numbers for the ITS sequences are: AY278557 (*P. anserina*), DQ336707 (*C. globosum*), DQ453701 (*F. graminearum*), AJ937756 (*A. nidulans*), DQ401534 (*A. fumigatus*) and DQ411551 (*A. oryzae*).
