# Supplementary material for: Genesis of a Fungal Non-Self Recognition Repertoire
Source: PLoS One. 2007 Mar 14;2(3):e283. doi: 10.1371/journal.pone.0000283 (PMC1805685; doi:10.1371/journal.pone.0000283)
Supplement: Figure S2 — Close up view of the P. anserina clade presented Fig. 2B. WD-40 repeats are designated by the gene of origin and their position from the first WD-40 repeat at the N-terminal end of the WD-repeat domain. Genes of origin are colour coded. Cyan branches indicate sequences associated to NACHT domains grouping in the N-I clade of the NACHT phylogeny. Internal Branch Length test values over 80 are indicated. (0.02 MB PDF) [file pone.0000283.s002.pdf]

Figure S2

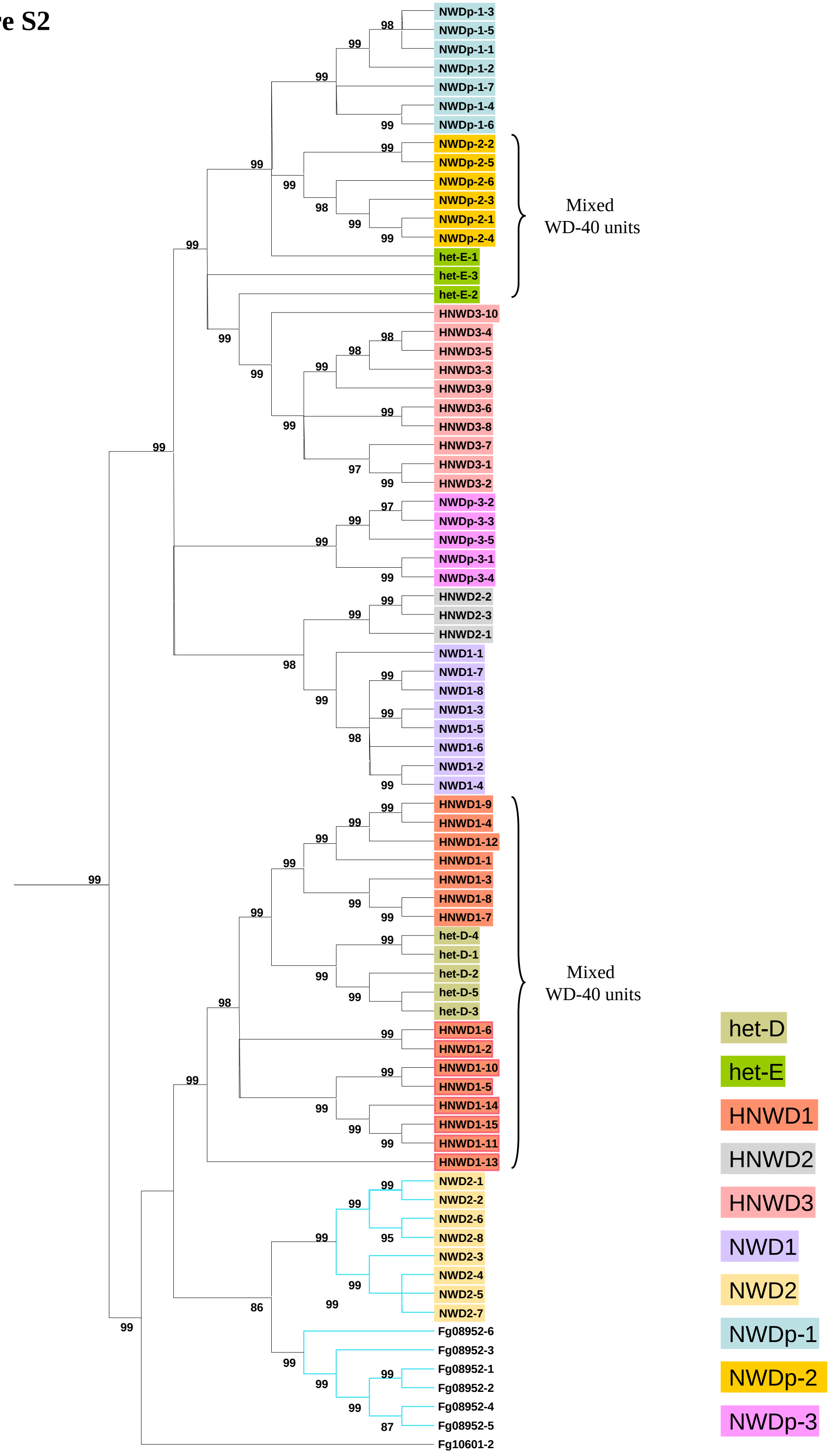

Figure S2: Close up view of the *P. anserina* clade presented Fig. 2B. WD-40 repeats are designated by the gene of origin and their position from the first WD-40 repeat at the N-terminal end of the WD-repeat domain. Genes of origin are colour coded. Cyan branches indicate sequences associated to NACHT domains grouping in the N-I clade of the NACHT phylogeny.Internal Branch Length test values over 80 are indicated.
