# Supplementary material for: Genesis of a Fungal Non-Self Recognition Repertoire
Source: PLoS One. 2007 Mar 14;2(3):e283. doi: 10.1371/journal.pone.0000283 (PMC1805685; doi:10.1371/journal.pone.0000283)
Supplement: Figure S3 — Full size WD-40 unit Neighbor-Joining phylogenetic tree identical to figure 2A. Loci of origin are noted, and species of origin are colour coded. Each WD-40 unit is designated by the gene of origin and the number of the WD-40 repeat from the N-terminal end of the domain. Cyan branches indicate sequences associated to NACHT domains grouping in the N-I clade of the NACHT phylogeny. Internal Branch Length test values over 80 are indicated. (0.02 MB PDF) [file pone.0000283.s003.pdf]

Figure S3

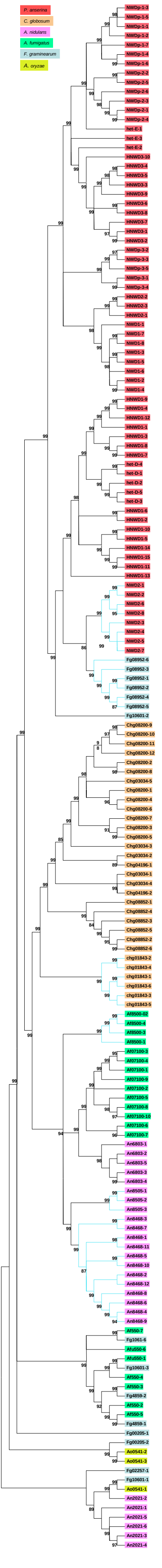

Figure S3: Full size WD-40 unit Neighbor-Joining phylogenetic tree identical to figure 2A. Loci of origin are noted, and species of origin are colour coded. Each WD-40 unit is designated by the gene of origin and the number of the WD-40 repeat from the N-terminal end of the domain. Cyan branches indicate sequences associated to NACHT domains grouping in the N-I clade of the NACHT phylogeny. Internal Branch Length test values over 80 are indicated.
