# Supplementary material for: Genesis of a Fungal Non-Self Recognition Repertoire
Source: PLoS One. 2007 Mar 14;2(3):e283. doi: 10.1371/journal.pone.0000283 (PMC1805685; doi:10.1371/journal.pone.0000283)
Supplement: Figure S4 — Maximum Parsimony phylogenetic trees constructed with the same data set as Neighbor-Joining tree reported Fig. 2. A/NACHT phylogeny, B/WD-40 phylogeny. Loci of origin are noted, and species of origin are colour coded. Each WD-40 unit is designated by the gene of origin and the number of the WD-40 repeat from the N-terminal end of the domain. Cyan branches of the WD-40 phylogeny indicate sequences associated to NACHT domains grouping in the N-I clade of the NACHT phylogeny. Bootstrap values over 50 are indicated. (0.03 MB PDF) [file pone.0000283.s004.pdf]

Figure S4

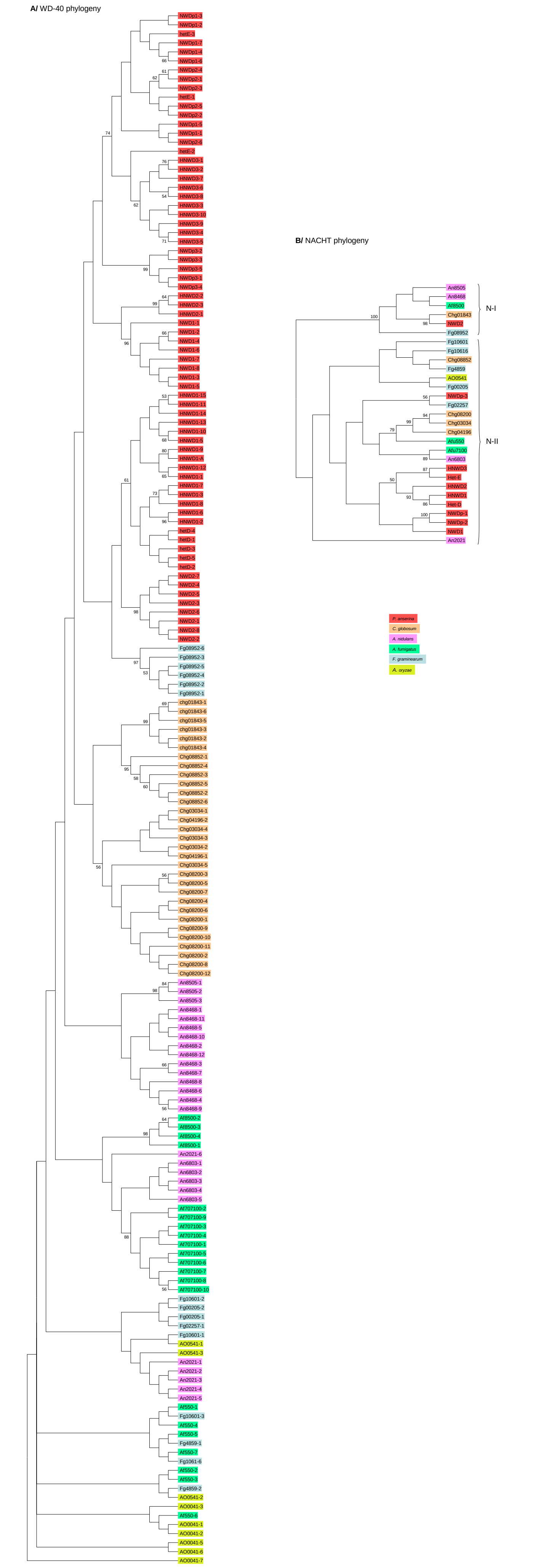

Figure S4: Maximum Parsimony phylogenetic trees constructed with the same data set as Neighbor-Joining tree reported Fig. 2. A/ NACHT phylogeny, B/ WD-40 phylogeny. Loci of origin are noted, and species of origin are colour coded. Each WD-40 unit is designated by the gene of origin and the number of the WD-40 repeat from the N-terminal end of the domain. Cyan branches of the WD-40 phylogeny indicate sequences associated to NACHT domains grouping in the N-I clade of the NACHT phylogeny. Bootstrap values over 50 are indicated.
