# Supplementary material for: Genesis of a Fungal Non-Self Recognition Repertoire
Source: PLoS One. 2007 Mar 14;2(3):e283. doi: 10.1371/journal.pone.0000283 (PMC1805685; doi:10.1371/journal.pone.0000283)
Supplement: Table S1 — Accession numbers of P. anserina NWD family genes Genes newly identified were named according to their structural composition. H = HET domain, N = NACHT domain, WD = WD-repeat domain. Pseudogenes are indicated by the suffixe p. (0.02 MB PDF) [file pone.0000283.s005.pdf]

Table S1: Accession numbers of *P. anserina NWD* family genes

Genes newly identified were named according to their structural composition. H = HET domain, N = NACHT domain, WD = WD-repeat domain. Pseudogenes are indicated by the suffixe p.

Gene name	Accession number
het-D (Espagne, Balhadere et al. 2002)	AF 323585, CU074316
het-E (Saupe, Turcq et al. 1995)	AF323581, AF323582, CU074315
HNWD1	CU074274
HNWD2	CU074273
HNWD3	CU074272
NWD1	CU074271
NWD2	CU074270
NWDp-1	CU062847
NWDp-2	CU062843
NWDp-3	CU062669
