# Supplementary material for: Genesis of a Fungal Non-Self Recognition Repertoire
Source: PLoS One. 2007 Mar 14;2(3):e283. doi: 10.1371/journal.pone.0000283 (PMC1805685; doi:10.1371/journal.pone.0000283)
Supplement: Table S2 — GC content of the NWD family GC content of the NWD loci were determined for each member of the gene family. Control values (CTL) were defined as the GC and TA contents averaged over twenty randomly chosen 750bp control sequences. Shaded values are significantly lower than the rest of the dataset at a 95% confidence interval. (0.02 MB PDF) [file pone.0000283.s006.pdf]

Table S2: GC content of the NWD family

GC content of the *NWD* loci were determined for each member of the gene family. Control values (CTL) were defined as the GC and TA contents averaged over twenty randomly chosen 750bp control sequences. Shaded values are significantly lower than the rest of the dataset at a 95% confidence interval.

	% GC	%AT
het-D	50.64	49.36
het-E	49.16	50.84
HNWD1	48.72	51.28
HNWD2	50.57	49.43
HNWD3	49.41	50.59
NWD1	55.55	44.45
NWD2	51.42	48.58
NWDp-1	43.85	56.15
NWDp-2	37.32	62.68
NWDp-3	51.36	48.64
CTL	50.23	49.77
