# Supplementary material for: Genesis of a Fungal Non-Self Recognition Repertoire
Source: PLoS One. 2007 Mar 14;2(3):e283. doi: 10.1371/journal.pone.0000283 (PMC1805685; doi:10.1371/journal.pone.0000283)
Supplement: Table S3 — Genes of the NWD family identified in other fungal species. (0.02 MB PDF) [file pone.0000283.s007.pdf]

Table S3 : Genes of the *NWD* family identified in other fungal species.

Species	Locus
A. fumigatus	Af550<br>Af7100<br>Af8500
A. nidulans	An8468<br>An8505<br>An2021<br>An6803
A. oryzae	AO090005000041<br>Abbreviated to Ao041<br>AO090701000541<br>Abbreviated to Ao541
C. globosum	Chg03034<br>Chg08200<br>Chg04196<br>Chg08852<br>Chg01843
F. graminearum	Fg02257<br>Fg4859<br>Fg00205<br>Fg10616<br>Fg10601<br>Fg8952
