# Supplementary material for: Genesis of a Fungal Non-Self Recognition Repertoire
Source: PLoS One. 2007 Mar 14;2(3):e283. doi: 10.1371/journal.pone.0000283 (PMC1805685; doi:10.1371/journal.pone.0000283)
Supplement: Table S4 — 55 combinations of amino acids at the positions under positive selection in the WD-40 repeats of the P. anserina NWD gene family. WD sequences are named as in figure 2. Identical combinations are colour shaded, and the number of different amino acids at each position is indicated (0.05 MB PDF) [file pone.0000283.s008.pdf]

Tale S4 : 55 combinations of amino acids at the positions under positive selection in the WD-40 repeats of the *P. anserina NWD* gene family. WD sequences are named as in figure 2. Identical combinations are colour shaded, and the number of different amino acids at each position is indicated

	Amino acid positions			
WD repeats	7	9	25	27
NWDp3-1	S	R	N	Q
NWDp3-4	S	R	N	Q
NWDp3-5	L	L	N	Q
NWDp3-2	S	R	D	G
NWDp3-3	W	R	D	G
NWD2-3	S	R	D	R
NWD2-7	S	R	D	R
NWD2-6	S	R	G	R
NWD2-8	S	R	G	R
NWD2-4	S	W	D	H
NWD2-5	S	W	D	R
NWD2-1	S	G	G	C
NWD2-2	W	W	G	R
hetD-3	W	N	D	H
hetD-4	P	N	D	H
hetD-1	S	N	D	H
hetD-2	W	L	A	S
hetD-5	W	Y	A	S
HNWD1-3	S	N	S	S
HNWD1-7	S	N	S	S
HNWD1-8	S	N	S	S
HNWD1-4.	S	N	G	D
HNWD1-9	S	N	G	D
HNWD1-14	F	L	R	K
HNWD1-15	W	M	R	K
HNWD1-1	P	D	R	K
HNWD1-5	S	M	Y	K
HNWD1-10	S	M	Y	K
HNWD1-2	W	K	D	S
HNWD1-6	W	K	D	S
HNWD1-11	W	K	G	K
HNWD1-12	S	H	G	K
HNWD1-13	S	M	N	K
HNWD3-6	T	R	V	E
HNWD3-7	S	R	V	N
HNWD3-3	P	L	V	K

HNWD3-10	S	L	V	K
HNWD3-4	P	W	V	K
HNWD3-5	P	W	V	K
HNWD3-9	T	W	V	K
HNWD3-8	P	W	V	E
HNWD3-1	P	R	D	N
HNWD3-2	P	W	D	N
hetE-2	S	W	I	G
NWDp1-1	S	W	L	K
NWDp1-5	S	W	L	M
NWDp1-4	W	Q	H	M
NWDp1-6	W	Q	H	K
NWDp1-2	W	Q	L	M
NWDp1-3	S	Q	V	K
hetE-1	S	L	G	K
NWDp2-1	L	L	H	N
NWDp2-4	L	L	H	N
NWDp2-3	L	W	H	N
NWDp2-6	S	W	H	N
NWDp2-2	S	L	S	R
NWDp2-5	S	L	D	R
hetE-3	W	H	S	N
HNWD2-2	S	Y	V	D
HNWD2-3	S	Y	V	C
HNWD2-1	S	Y	G	R
NWD1-2	W	S	D	R
NWD1-4	W	S	D	R
NWD1-3	L	M	D	R
NWD1-1	S	R	D	R
NWD1-7	W	R	G	E
NWD1-8	W	M	G	E
NWD1-5	L	M	G	K
NWD1-6	W	R	H	K
Total amino acids	6	12	11	11
