# Supplementary material for: Genesis of a Fungal Non-Self Recognition Repertoire
Source: PLoS One. 2007 Mar 14;2(3):e283. doi: 10.1371/journal.pone.0000283 (PMC1805685; doi:10.1371/journal.pone.0000283)
Supplement: Table S5 — Mutation frequencies at silent positions in the WD-40 sequences. WD-40 sequences were analysed as two pools according to their belonging to one of the two main clades in the WD40-phylogeny presented figure 2. One pool comprised WD-40 sequences from loci NWD-1, het-E, NWDp1, NWDp2, NWDp3 and HNWD3, the other pool comprised sequences from loci NWD2, Het-D, HNWD1 and HNWD2. C to T transitions in the pool of WD-40 sequences compared to the consensus sequences were counted on both strands and are reported according to their context. (0.02 MB PDF) [file pone.0000283.s009.pdf]

Table S5 : Mutation frequencies at silent positions in the WD-40 sequences.

WD-40 sequences were analysed as two pools according to their belonging to one of the two main clades in the WD40- phylogeny presented figure 2. One pool comprised WD-40 sequences from loci *NWD-1*, *het-E*, *NWDp1*, *NWDp2*, *NWDp3* and *HNWD3*, the other pool comprised sequences from loci *NWD2*, *Het-D*, *HNWD1* and *HNWD2*. C to T transitions in the pool of WD-40 sequences compared to the consensus sequences were counted on both strands and are reported according to their context.

	Context			
Mutations	CpA	CpT	CpG	CpC
C to T transitions	7 (78%)	6 (42%)	7 (38%)	4 (44%)
C to A transversions	0 (0%)	1 (7%)	3 (16%)	0 (0%)
C to G transversions	1 (11%)	3 (21%)	6 (33%)	1 (11%)
Conserved	1 (11%)	4 (28%)	2 (11%)	4 (44%)
Total	9	14	18	9
