# Supplementary material for: Genesis of a Fungal Non-Self Recognition Repertoire
Source: PLoS One. 2007 Mar 14;2(3):e283. doi: 10.1371/journal.pone.0000283 (PMC1805685; doi:10.1371/journal.pone.0000283)
Supplement: Table S6 — Primers used to PCR amplify WD repeat domains from P. anserina NWD gene family members. (0.02 MB PDF) [file pone.0000283.s010.pdf]

Table S6 : Primers used to PCR amplify WD repeat domains from *P. anserina NWD* gene family members.

locus	Forward primer	Reverse primer
het-D	GCTTCAGCACTTGTGTTTAGC	CGTTAGATCCAGATGCAATCAGC
het-E	GGCTGGTCTAACAGTAGTATAGG	GTGTACAGGTTCCCGATGCC
HNWD1	GAACGGAAGTGGATTGCTTTGG	CCAGACCCCTCCTGTGTCACG
HNWD2	GCACCGGTTGGCAGTCTGG	CCAGGCCCTTCTCGTGTTAGG
HNWD3	GTACGGGATCAGTAGAAATGC	CACCCCTTTGGTGGTAAACG
NWD1	CCTCATTAGAGAACTCTTCAAGAAGG	CTACTGAGAACTAAAAAGGGCTGC
NWD2	GTACAAGATAGTTGGAATGCGTGC	CCCCTCCTTTGGCACGCTACG
NWDp-1	CCTTATATTTATACCAAGTAGCAGC	GTATAGTCGAACGATAGATAGG
NWDp-2	GGACGGCCAGCGCGTGG	CCAGATCTTGATGGTATTATCG
NWDp-3	GTCGTCGAGCAAAACTGGAGTCC	CCTTCGAGCTTCCGTACCTCC
